# New Nonlinear Active Element Dedicated to Modeling Chaotic Dynamics with Complex Polynomial Vector Fields

**DOI:** 10.3390/e21090871

**Published:** 2019-09-06

**Authors:** Jiri Petrzela, Roman Sotner

**Affiliations:** Department of Radio Electronics, Faculty of Electrical Engineering and Communication, Brno University of Technology, 616 00 Brno, Czech Republic; sotner@feec.vutbr.cz

**Keywords:** bifurcation diagram, chaotic oscillator, Lyapunov exponents, polynomial vector field, squarer, trans-conductance mode

## Abstract

This paper describes evolution of new active element that is able to significantly simplify the design process of lumped chaotic oscillator, especially if the concept of analog computer or state space description is adopted. The major advantage of the proposed active device lies in the incorporation of two fundamental mathematical operations into a single five-port voltage-input current-output element: namely, differentiation and multiplication. The developed active device is verified inside three different synthesis scenarios: circuitry realization of a third-order cyclically symmetrical vector field, hyperchaotic system based on the Lorenz equations and fourth- and fifth-order hyperjerk function. Mentioned cases represent complicated vector fields that cannot be implemented without the necessity of utilizing many active elements. The captured oscilloscope screenshots are compared with numerically integrated trajectories to demonstrate good agreement between theory and measurement.

## 1. Introduction

The synthesis and practical realization of chaotic deterministic dynamical systems in the form of lumped electronic circuits is an old and well-established problem. Several design methods have already been described and verified in journal papers as well as conference contributions. The rest of this section provides detailed insight into various circuitry realizations of chaotic oscillators. We will focus especially on the problems of implementing complex nonlinear mathematical models; that is, differential equations having many terms and scalar nonlinear functions of the polynomial shape.

The very first concepts of the analog chaotic oscillators were designed to be simple and transparent, in order to demonstrate the fundamental nature of chaos and the conditions that are required for the evolution of chaotic behavior. These circuits can be considered as a parallel connection of the higher-order admittance network and nonlinear active two-terminal device. This resistor can be piecewise linear; for example, where a third-order admittance network contains both capacitors and inductors as functional accumulation elements is provided in [1], while an RC passive ladder network connected as a load for active nonlinear resistors is the primary subject of [2]. Polynomial resistors with arbitrary degrees [3] can be connected in parallel with a third-order fully passive admittance network to obtain a robust chaotic oscillator [4,5] as well. Moreover, higher-order polynomial resistors can be used to approximate goniometric functions and connected as the fundamental nonlinearity inside suitable circuit topology to generate the so-called multiscroll [6,7,8] or multigrid strange attractors [9,10]. By generalization of such a design approach, very simple chaotic systems with a fully passive ladder filter working in the trans-immittance regime can be constructed [11]. So far, it seems that this is also the simplest way to practically implement the so-called jerky (motion or Newtonian) dynamics, i.e., autonomous deterministic system defined by a single third-order ordinary differential equation [12,13,14,15]. Here, a trans-immittance passive ladder filter having low-pass type of frequency response is connected in the closed loop with the active nonlinear two-port. Thus, the total number of circuit elements can be theoretically reduced to four. For a trans-immittance filter with low quality factor (dynamical system with the higher dissipation), the RLC structure can be substituted by an RC equivalent.

Concepts of fully analog oscillators with a nonlinear composition generated by passive-only nonlinear devices, such as diodes or transistors, timing network, and simple active energy source, also belong to the simplest robust chaotic systems [16,17]. Of course, second-order two-terminal devices (both ideal and lossy) can be used for energy distribution toward the rest of circuitry [18,19]. Chaotic oscillators can be also designed by a slight modification of the well-known generators of harmonic waveforms. Such a modification is often based only on inclusion of suitable nonlinear circuit element into common circuitry [20,21]. 

If second-order-driven dynamical systems become included in our considerations, the state variable filters are also examples of jerky dynamics. Recent discoveries reveal that robust chaos can be observed in the common structures of Kerwin-Huelsmann-Newcomb (KHN) filters [22,23] if some sort of intrinsic saturation-type nonlinearity is respected. Of course, chaos can be intentionally generated by non-autonomous dynamical systems. The simplest of such an example can be found in a short study [24] where the second-order-driven dynamical system only has three terms, including the scalar non-proportional relation of a state variable. Forced chaotic oscillators can be knowingly constructed as nicely demonstrated in [25,26]. The evolution of chaos is conditioned by the shape of the harmonic input variable, i.e., amplitude and/or frequency. This means that one of these basic parameters of the input signal can be used as the natural bifurcation parameter and serve to control some route-to-chaos scenario. Thus, new network components that can be marked as analog-to-chaos converter can be constructed. Two types become available depending on the input variable (output is always voltage); in the case of voltage, we are referring to the two-port chaos converter [27], and for current input variable, we are dealing with a two-terminal chaos converter [28], i.e., a chaotic impedance. So far, despite its robustness, the described network elements are still awaiting practical application.

A systematic approach, that is the most often utilized for the design of chaotic oscillators based on a mathematical model, follows the well-known concept of analog computers. Individual first-order ordinary differential equations are implemented by using summing inverting integrators with single voltage-feedback operational amplifier. If required, additional summation and/or subtraction linear operations are realized by a single operational amplifier-based differential amplifier. Final building blocks bring the folding mechanism into vector field geometry; nonlinear two-ports are constructed with prescribed voltage transfer function. Now, it is the right time to recall a few interesting chaotic oscillators that utilize the method mentioned above and provide some unique features. Circuitry realization of the complex butterfly chaotic attractors having multiple wings can be found in [29,30], and a multiscroll strange attractor is implemented in an overview paper [31]. Here, curious reader can compare several design methods from different perspectives. We can also consider systematic design procedures toward multispiral chaotic attractors with user-defined complexity of the piecewise-linear transfer function; see details in [32]. Described integrator-based block method is universal and can be utilized (without changes) for design of the higher-order dynamical systems. For example, a four-dimensional oscillator is the subject of [33]. The discussed integrator-based circuit synthesis is summarized and turned into a systematic design process in a comprehensive study [34]. In the case of nonlinear transfer functions having very complicated shapes, corresponding two-port blocks can be implemented by using digital parts, i.e., via combination of a simple processor and by using a lookup table approach, and A/D and D/A converters. Remember that this substitution turns a chaotic system into the piecewise constant. However, global dynamics can be preserved [35]. The recent trend is to utilize field programmable analog arrays for complete design of nonlinear dynamical systems. This is advantageous due to the fast design and easy reconfiguration of topology of the chaotic oscillators [36]. On the other hand, chaotic oscillators can be implemented as systems-on-chip using uniform fabrication technology. Both advantages and limitations of such analog realizations can be found in interesting research studies [37,38].

All integrator-based designs have something in common, namely, a complicated structure with many active and passive circuit elements. The aim of this paper is to partly remove this drawback and slightly simplify the final circuit topology. As authors, we have to admit that this effort is not unique. Chua’s original circuit was simplified by adopting different active devices, such as a current-feedback operational amplifier with frequency compensation in [39] or, alternatively, by a differential voltage current conveyor as given in [40]. The monolithic implementation of Chua’s circuit is the subject of [41]. Negative resistance chaotic oscillators can be effectively constructed by using a second generation current conveyor [42], by standard voltage-feedback operational amplifier [43], as well as any other active element, including a two-terminal structure containing only few discrete FET components [44]. This paper contributes to this kind of research by introducing a versatile multiport active element suitable for modeling nonlinear dynamics.

This paper is organized as follows. The next section briefly describes layout design of a new active device and also contains measurement results of its key properties from the viewpoint of modeling nonlinear dynamics. The third section is focused on the presentation of several mathematical models that undergo true experimental verification. Within this section, the fundamental analysis of these models based on the numerical integration process is presented. The fourth section brings circuitry realizations of chaotic systems, where both linear and nonlinear mathematical operations are performed by a new active element. The fifth section provides us with measurement results and comparison between theoretical and practical experiments. Finally, some discussion and concluding remarks are presented.

## 2. Evolution of Differential Voltage Trans-Conductance Multiplier (DV-TC-M)

**Figure 1:**
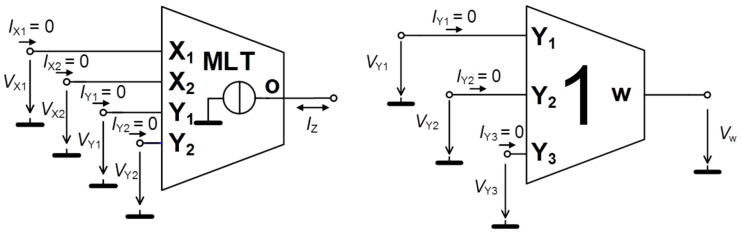
Symbols of active cells employed in designed chaotic oscillators: multiplier with internal voltage-to-current conversion (**left**) and summation/subtraction (**right**) unit.

The new active cells, DV-TC-M, were preliminarily tested in non-problematic applications [45,46] and will be utilized in the structures of fully analog chaotic oscillators presented in this paper. Layout designs of the individual developed functional blocks are visualized inside Appendix A. Idealized models of these structures are provided in Figure 1, and fabricated examples are based on I3T25 0.35 µm ON Semiconductor CMOS process that comprised a single integrated circuit (IC) package. The trans-conductance transfer function of device is available in two equivalent forms but with different value of constant *k* depending on the internal topology:

(1) $$k\cdot \left(V_{X1}-V_{X2}\right)\cdot \left(V_{Y1}-V_{Y2}\right)=I_{Z},$$

where *k* = 4.9 mA/V^2^ for a bipolar multiplier and *k* = 1.3 mA/V^2^ for a CMOS multiplier. Note that such transfer function is versatile especially in the case where individual ordinary differential equations are implemented by directly following Kirchhoff’s first law; that is, the sum of the currents associated with a single node equals zero. Summation/subtraction building block performs a useful operation, returning the sum and subtraction of three input voltages in accordance to the following equation:

(2) $$V_{Y1}-V_{Y2}+V_{Y3}=V_{W}.$$

**Figure 2:**
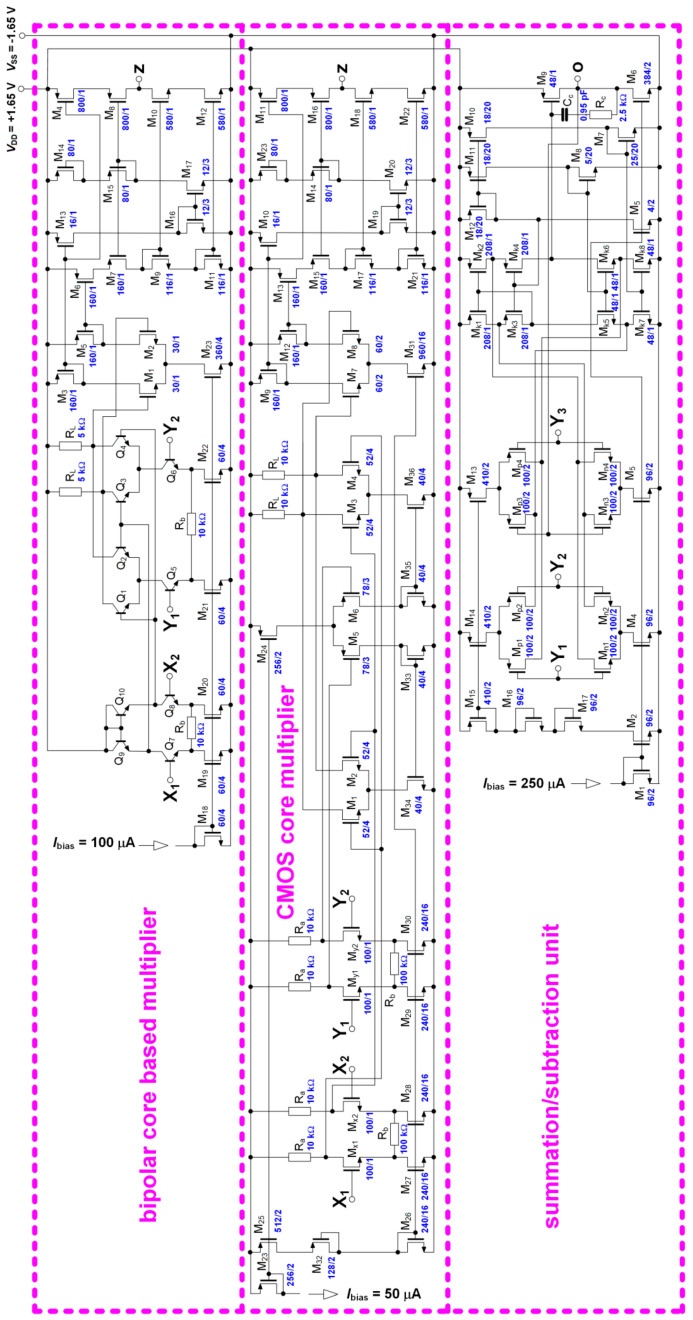
Internal topologies of the active cells fabricated in a single integrated circuit (IC) package.

A complete view of the internal topologies of the active cells is shown in Figure 2, including W/L ratios of FET transistors and values of resistors. 

**Figure 3:**
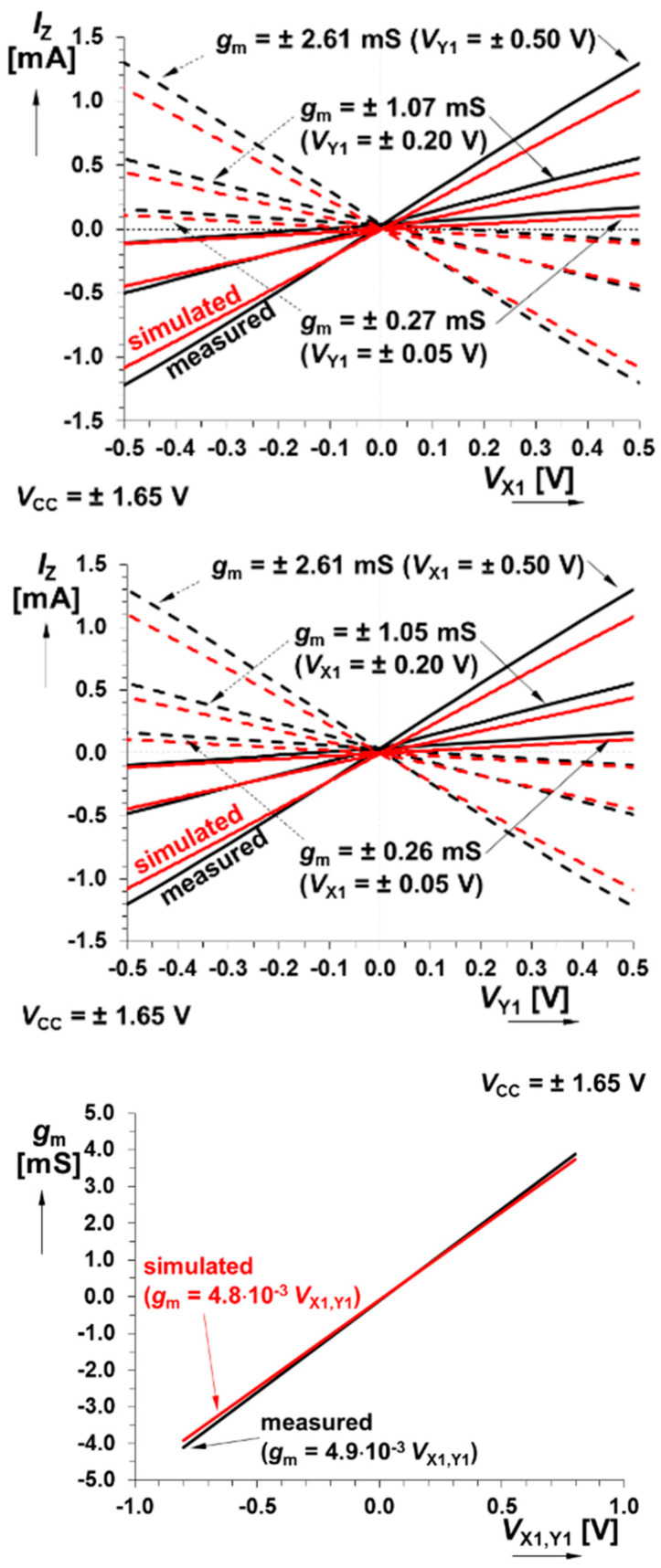
Comparison between measured and simulated responses of bipolar core-based multiplier: DC transfer responses X_1_→Z for *V*_Y1_ controlled by DC voltage (**upper plot**), DC transfer responses Y_1_→Z for *V*_X1_ controlled by DC voltage (**middle plot**), and dependence of *g*_m_ on *V*_Y1_/*V*_X1_ (**lower plot**).

DC transfer curves belong to the most important characteristics of the active devices employed in the design process of chaotic oscillators and linear and nonlinear systems in general. Figure 3 shows that transfer linearity is good over the entire allowed dynamical range of the input voltages. The measured features of the bipolar multiplier predetermine this cell for all applications that operate with input voltages of ±500 mV (this limits the state space volume occupied by a constructed chaotic attractor). There is only linear distortion that can be easily compensated by the external circuitry, in practice, by simple component value recalculation. The maximal allowed output current is 1500 mA.

To make the electronic equivalents precise for the prescribed mathematical models, we should perform accurate operations such as squaring and multiplication. Note that amplification/attenuation can be considered as the product of a state variable and DC constant voltage. Thus, it also belongs to this class of scalar mathematical operations. 

**Figure 4:**
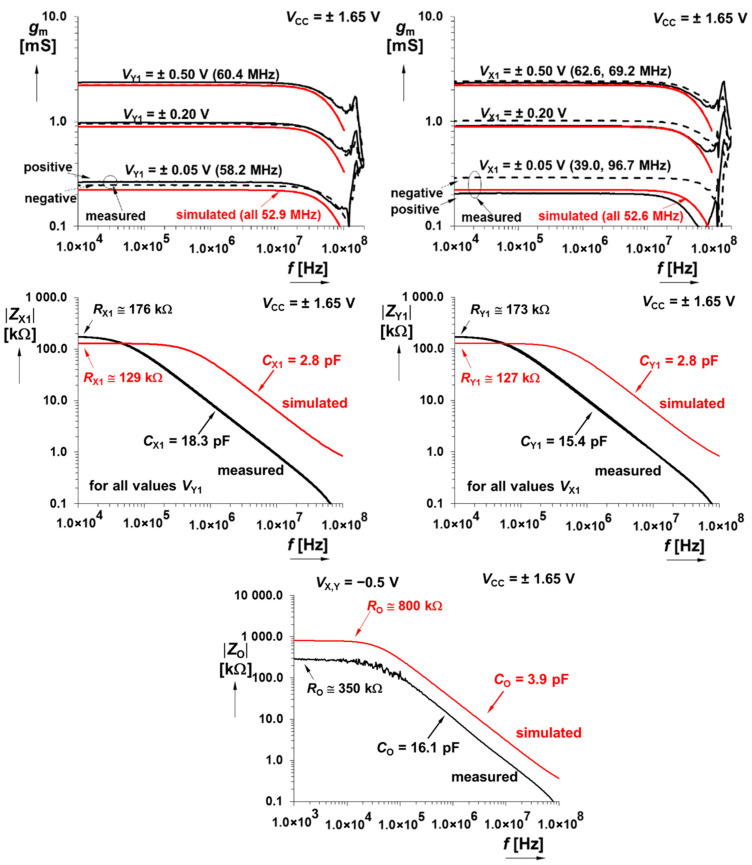
Magnitude of AC transfer responses X_1_→Z for *V*_Y1_ controlled by DC voltage (**upper left plot**), magnitude of AC transfer responses Y_1_→Z for *V*_X1_ controlled by DC voltage (**upper right graph**), magnitude vs. frequency plot of input impedance at X_1_ terminal (**middle left plot**), magnitude vs. frequency plot of input impedance at Y_1_ terminal (**middle right plot**), and magnitude vs. frequency plot of output impedance at Z terminal, example for *V*_X,Y_ = −0.5 V (**lower plot**).

Since all chaotic signals are naturally wideband, module frequency responses of active devices involved in a design process should be maximally horizontally flat. In this context, Figure 4 shows that DV-TC-M has a bandwidth 50 MHz; i.e., sufficiently wide for any type of strange attractor.

Wide bandwidth is very important because roll-off effects can filter out the frequency components required to form a desired strange attractor. This value should also be respected when choosing a frequency de-normalization constant. A nice case study has focused on the current feedback operational amplifiers that can be found in [47].

**Figure 5:**
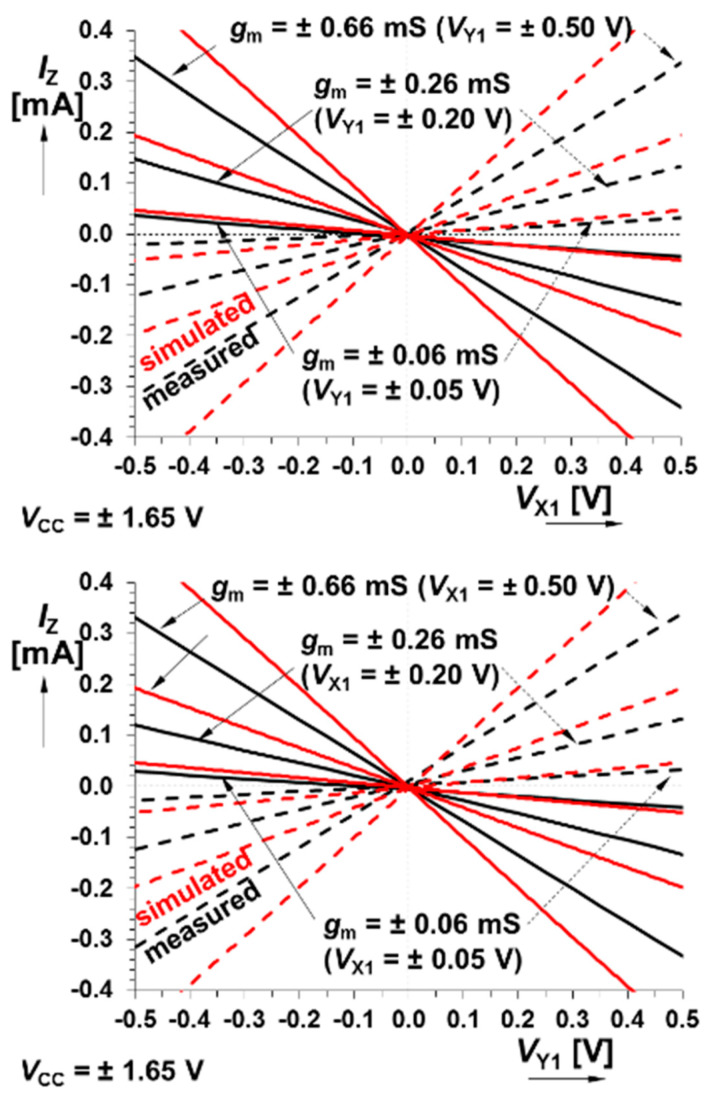

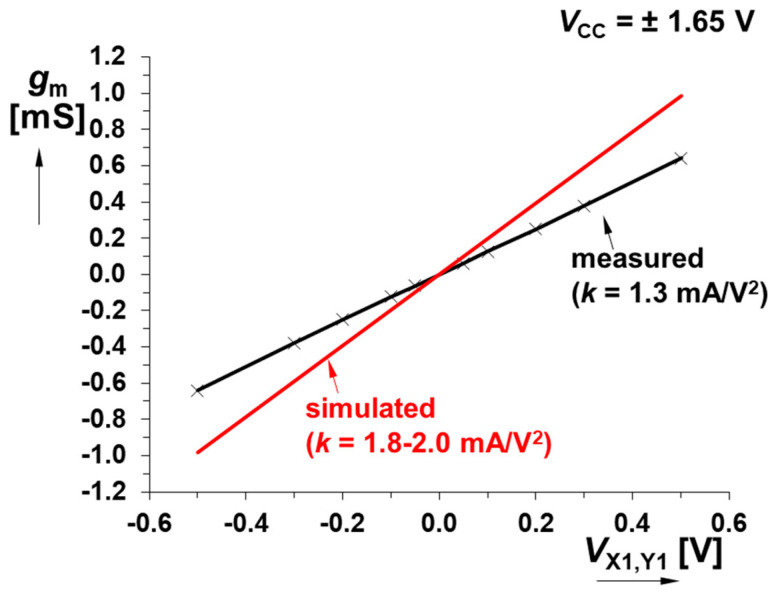
Comparison between measured and simulated responses of CMOS core-based multiplier: DC transfer responses X_1_→Z for *V*_Y1_ controlled by DC voltage (**upper image**), DC transfer responses Y_1_→Z for *V*_X1_ controlled by DC voltage (**middle plot**), and dependence of *g*_m_ on *V*_Y1,X1_ (**lower image**).

**Figure 6:**
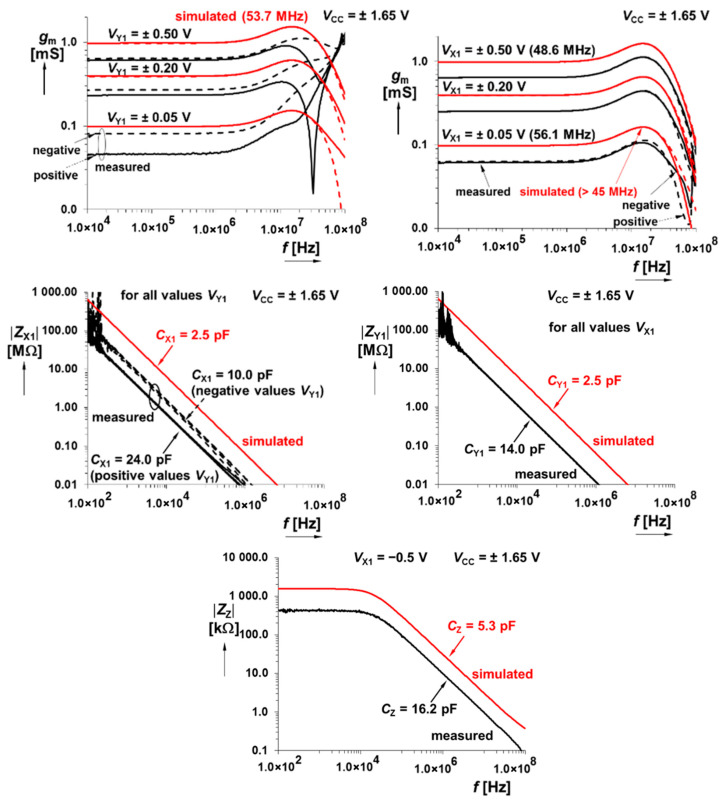
Measured magnitude of AC transfer X_1_→Z responses for *V*_Y1_ controlled by DC voltage (**upper left plot**), magnitude of AC transfer responses Y_1_→Z for *V*_X1_ controlled by DC voltage (**upper right plot**), magnitude vs. frequency plot of input impedance at X_1_ node (**middle left image**), magnitude vs. frequency plot of input impedance at Y_1_ node (**middle right plot**), and magnitude vs. frequency plot of output impedance at Z terminal, example for *V*_X1_ = −0.5 V (**lower graph**).

**Figure 7:**
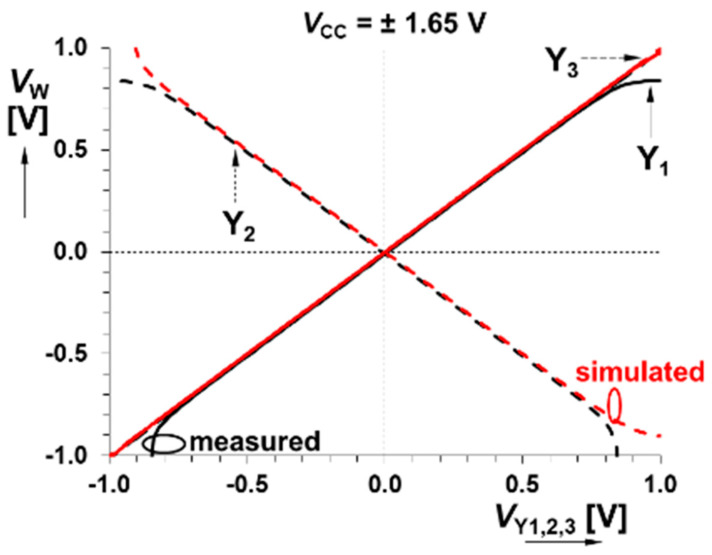
Comparison between measured and simulated responses of a summation/subtraction unit (see text for evaluation): DC transfer responses between terminals Y_1,2,3_→W.

**Figure 8:**
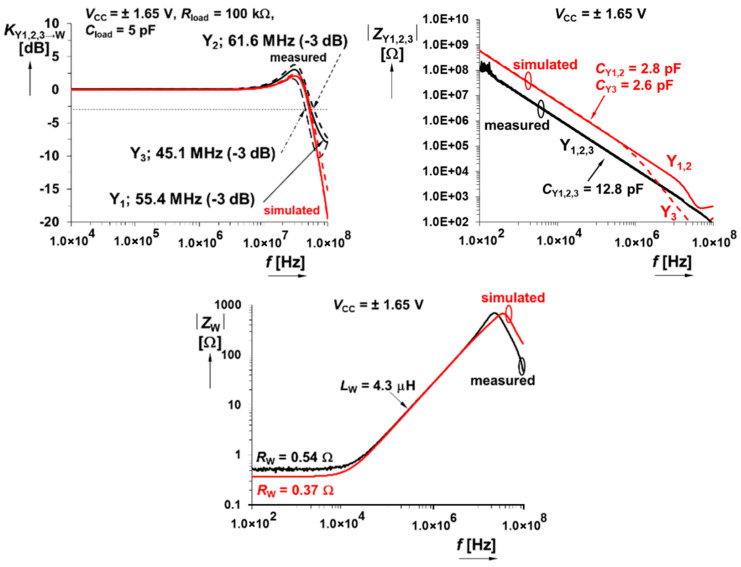
Summation/subtraction unit and magnitude AC transfer responses between terminals Y_1,2,3_→W (**upper left plot**), magnitude of the input impedances Y_1,2,3_ vs. frequency (**upper right graph**), and magnitude vs. frequency plot of output impedance measured at W terminal (**lower image**).

The CMOS multiplier has an allowed level of input signal up to ±500 mV, and the bandwidth reaches at least 30 MHz. Further interesting (from the viewpoint of modeling nonlinear dynamics) measured results are documented via Figure 5 and Figure 6. Summation/subtraction unit allows operation of useful signals up to ±700 mV and 3 dB bandwidths of transfers overcome 45 MHz. For details, see the graphical visualization given in Figure 7 and Figure 8.

## 3. Numerical Analysis of Chaotic Systems Dedicated for Circuit Synthesis

Before starting a design process of the analog chaotic oscillator, its dynamics need to be deeply analyzed by using computer algorithms that are mostly based on the numerical integration process. Individual upcoming subsections provide numerical investigation of several mathematical models. The analysis provided here is primarily focused on the upcoming circuit realization of dynamical systems. The algorithms that are utilized here can be summarized as follows.

(1) Largest Lyapunov exponent (LLE). It is a real number that specifies the rate of exponential divergence of two neighboring state trajectories. For chaos, it is necessary to achieve one positive LLE, while for hyperchaos, we need two positive LLEs. This measure is plotted with respect to single or several system parameters which can change the global behavior of an analyzed circuit. A high value of LLE can be understood as dynamical motion that is unpredictable for short times. Transient behavior is always omitted such that calculation starts on the *ω*-limit set. During each routine step, Gram–Schmidt orthogonalization is performed. LLE can be calculated by using either a mathematical model [48] or data sequences generated by the system under inspection [49]. Subsequently, this measure can be used for flow qualification or optimization [50]. Rules for correct calculation of LLE are specified in [51].

(2) Approximate entropy (AE) is a real number that can quantify degree of similarity between consecutive patterns of the waveforms generated in a time domain. A significantly high value of AE suggests possible application of analyzed continuous-time chaotic signal in secure communications, chaos-based modulation, or masking of the useful analog signal. AE is established for each designed chaotic oscillator using real measured data sequences.

(3) Basins of the attraction (BoA) are subspaces of the state space volume that specify attraction sets for individual types of *ω*-limit sets. Possible types of attraction sets, including hidden chaotic attractors, need to be calculated by appropriate algorithms. It is important from both a theoretical and practical viewpoint. If the evolution of some specific state attractor is bounded to a specific set of initial conditions, these need to be imposed into an oscillator by suitable circuitry. Fortunately, for the proposed voltage-mode structures of a chaotic system, one or several capacitors can quite easily be pre-charged at any time.

(4) One-dimensional bifurcation diagram (BD) can be very useful for tracing the ideal routing-to-chaos scenario with continuous change of a single system parameter; especially if a very small step size of swept parameter is used. This graph is commonly considered as suitably chosen Poincare sections (subspaces of the state space) plotted and arranged next to each other. 

The numerical algorithms mentioned above were implemented in Mathcad 15 program; to be more specific, a built-in fourth-order Runge–Kutta method with a fixed time step was employed as the core process, i.e., for numerical integration. Since chaotic dynamics is expected, possible problems with conventional numerical algorithms can be expected: such as convergence, chaos suppression, finite precision of real numbers, etc. In other words, credibility of the results may be compromised; as pointed out in [52]. For example, too large step sizes used for integration process can cause chaos suppression. Thus, only higher-order integration methods with a step size several orders lower than the lowest time constant of the designed chaotic circuit were considered for analysis (LLE, AE, BoA, BD). The second observed problem is related to finite precision of floating-point numbers. This will cause final periodicity of all chaotic orbits under one condition: if these are integrated for an enormously long time; however, this is not the case. The examples of chaotic flows considered in this paper are obviously durable against rounding errors [53]. Vector fields are smooth and continuously differentiable with fast trajectory stretching and transparent trajectory folding mechanisms. All parameters required for numerical re-simulation of results will be provided in the proper places in the text to ensure repeatable analysis. Closer insight into the analysis of autonomous deterministic chaotic dynamical systems and the problems of flow discretization, including corresponding solutions, can be found in a comprehensive study [54]. Although this paper warns readers to rely solely on the computer-aided results, especially if a chaotic dynamic is involved, we can trust our simulations are rigorous because these are verified by true experimental measurements.

Results from the numerical analysis are closely bounded to concrete circuit implementation and the real circuit parameters. This means that describing mathematical models are not normalized; i.e., neither time constants nor impedance scaling factors are introduced.

### 3.1. Numerical Investigation of Dynamical System with Cyclically Symmetrical Vector Field

**Figure 9:**
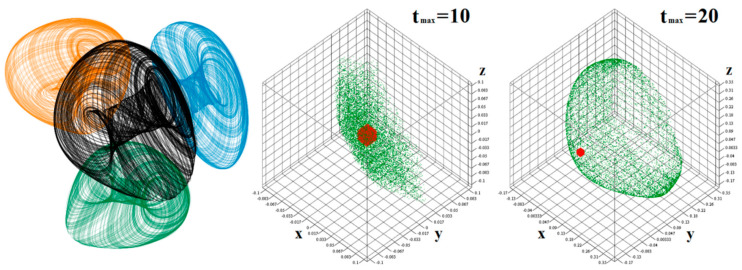
Three-dimensional perspective view on the typical strange attractor associated with the dynamical system (3) (**black color**) and corresponding plane projections: *x* vs. *y* (**green**), *x* vs. *z* (**blue**) and *y* vs. *z* (**orange**) for initial conditions **x**_0_ = (0.1, 0, 0)^T^. Sensitivity of the dynamical system (3) to initial conditions for different final time of integration; see text for clarification.

So far, several autonomous chaotic dynamical systems with cyclically symmetrical vector field and associated chaotic behavior were discovered [55,56]. One such example can be mathematically described by the following set of ordinary differential equations

(3) $$\frac{d}{dt}x=a\cdot y^{2}-b\cdot z\;\;\;\;\;\;\;\;\;\;\frac{d}{dt}y=a\cdot z^{2}-b\cdot x\;\;\;\;\;\;\;\;\;\;\frac{d}{dt}z=a\cdot x^{2}-b\cdot y,$$

where *a* and *b* are system parameters. To observe robust strange attractor, these parameters should be set to *a* = 1.9 and *b* = 0.42. Straightforward linear analysis yields a fixed point located at **x**_0_ = (0, 0, 0)^T^ and **x**_0_ = (*b*/*a*, *b*/*a*, *b*/*a*)^T^. Phase portrait of a typical chaotic attractor together with the measure of its sensitivity to tiny changes in the initial conditions is provided as in Figure 9. Sensitivity graphs are plotted for 10^4^ randomly generated initial conditions that are uniformly distributed in all three state space dimensions to create a filled cube with edge size 0.01 (red dots). Final states are marked by a green color. Note that the dynamical system is clearly extremely sensitive to tiny changes in initial conditions. The desired strange attractor occupies a state space cube with edges −0.2 up to 0.3 in all directions, i.e., for all state variables. Thus, the essential condition for a dynamical system to be implementable by using DV-TC-M active elements is satisfied. However, the larger state attractor can be also modeled by circuitry with DV-TC-M, as long as suitable linear transformation of the coordinates is applied to the original system that makes the state attractor smaller.

**Figure 10:**
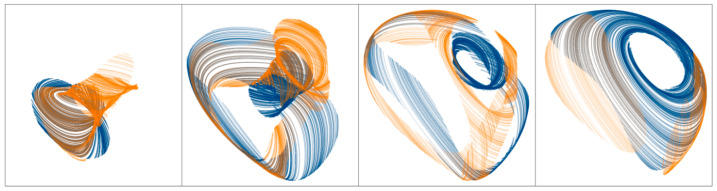
Energy distribution over strange attractor (see text): static (**blue**) and dynamic (**orange**).

Figure 10 provides rough insight into the energy distribution over the typical strange attractor generated by a dynamical system (3) and for standard values of the internal parameters. From the left to the right plot, one can see how potential and kinetic energy changes from very small (left picture), small, medium up to high (right plot).

**Figure 11:**
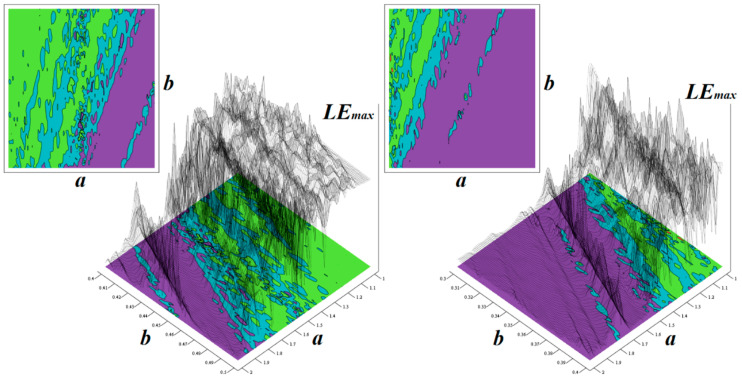
2D and 3D view of the rainbow color-scaled surface contour plot of the largest Lyapunov exponent (LLE) as a function of the internal system parameters *a* ∈ (1, 2) with step size 0.01 and *b* ∈ (0.3, 0.5) with step size 0.001.

Note that kinetic energy is negligible near the state space origin and large at the perimeter of a chaotic attractor. During simulations, the final time was set to 5000 s with time step 10 ms and initial conditions were chosen as **x**_0_ = (0.1, 0, 0)^T^. The static system energy has a direct connection to the power dissipation of chaotic oscillator while the dynamic energy is bounded to a slew rate and measured frequency responses (pole frequencies, GBP in particular) of used active elements. Figure 11 shows a plot of LLE associated with a mathematical model (3) as two-dimensional function of the internal system parameters. Here, various limit cycles correspond to magenta while light blue stands for weak chaos, green represents strong chaos, and red means unbounded solution. During calculations, the final time was set to 1000 s with variable time step and the initial conditions **x**_0_ = (0.1, 0, 0)^T^. In the optimal circumstances, a dynamical system can possess the following promising set of three Lyapunov exponents 0.106, 0, −0.967 and corresponding fractal Kaplan–Yorke dimension of about 2.11. Note that, in a given two-dimensional space of system parameters, the region of chaos is wide enough for practical construction of a robust generator of chaotic waveform and surrounded by a limit cycle solution.

### 3.2. Numerical Analysis of Fourth-Order and Fifth-Order Jerky Dynamics

Recent studies [57,58] reveal the possibility to observe robust strange attractors within the dynamics of fourth-, fifth-, and even higher-order differential equations with many types and shapes of scalar nonlinearity, including quadratic polynomials. Such dynamical flows are usually coined in literature as hyperjerk systems. These *n*-th order systems can be rewritten in the form of *n* first-order ordinary differential equations and implemented in developed numerical algorithms 1–4 without changes. As will be proved in this subsection, these mathematical models can exhibit a large variety of dense strange attractors.

**Figure 12:**
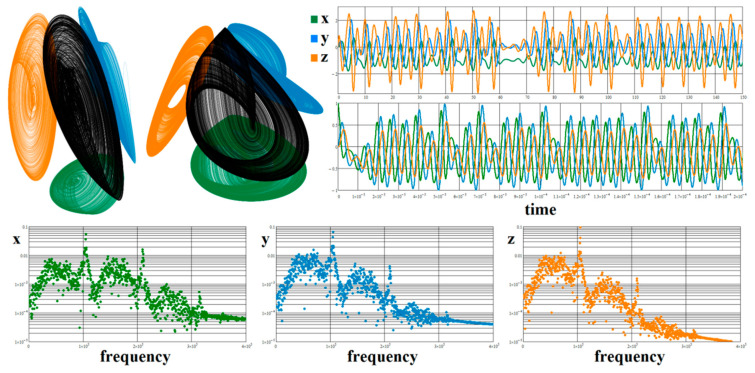
Perspective view on a typical strange attractor associated with system (4) is demonstrated in the left plot and circuit-oriented equivalent flow (10) is provided on the middle plot. The next pictures give generated chaotic waveforms in time and frequency domain for normalized and real circuit.

General circuitry realization of a fourth-order polynomial hyperjerk function can be described by the following ordinary differential equation:

(4) $$\frac{d^{4}}{dt^{4}}x+a\frac{d^{3}}{dt^{3}}x+b\frac{d^{2}}{dt^{2}}x+c\frac{d}{dt}x=d\cdot \left(x^{2}-1\right),$$

where *x* is some network quantity (depending on circuit realization) and *a, b, c*, *d* are internal system parameters, i.e., real numbers. There are just two equilibrium points of the flow located in position **x**_e_ = (±1, 0, 0, 0)^T^. To observe structurally stable strange attractors, the following values need to be kept: *a* = 1, *b* = 5.2, *c* = 2.7, and *d* = 4.5. For these numerical values, a typical single-scroll strange attractor provided in Figure 12 can be observed. Here, a numerical FFT calculation for the real circuit is also provided.

The fifth-order jerky dynamical system that produces robust chaotic motion can be expressed as a single differential equation:

(5) $$\frac{d^{5}}{dt^{5}}x+a\frac{d^{4}}{dt^{4}}x+b\frac{d^{3}}{dt^{3}}x+c\frac{d^{2}}{dt^{2}}x+d\frac{d}{dt}x=e\cdot \left(x^{2}-1\right).$$

**Figure 13:**
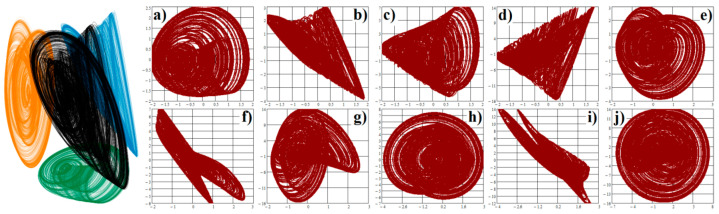
Three-dimensional perspective view (black) of the typical strange attractor associated with system (5) is provided in the left plot: *x* vs. *dx*/*dt* (green), *x* vs. *dx*^2^/*dt*^2^ (blue) and *x* vs. *dx*^3^/*dt*^3^ (orange). Remaining images represent the individual plane projections of a fifth-dimensional strange attractor: (**a**) *x* vs. *dx*/*dt*, (**b**) *x* vs. *d*^2^*x*/*dt*^2^, (**c**) *x* vs. *dx*^3^/*dt*^3^, (**d**) *x* vs. *dx*^4^/*dt*^4^, (**e**) *dx*/*dt* vs. *dx*^2^/*dt*^2^, (**f**) *dx*/*dt* vs. *dx*^3^/*dt*^3^, (**g**) *dx*/*dt* vs. *dx*^4^/*dt*^4^, (**h**) *dx*^2^/*dt*^2^ vs. *dx*^3^/*dt*^3^, (**i**) *dx*^2^/*dt*^2^ vs. *dx*^4^/*dt*^4^, (**j**) *dx*^3^/*dt*^3^ vs. *dx*^4^/*dt*^4^.

To generate the strange attractor, individual system parameters should be set to *a* = 1, *b* = 7.2, *c* = 3.9, *d* = 9.2, and *e* = 3.9. Similarly as for system (4), there are just two equilibrium points of the flow located in position **x**_e_ = (±1, 0, 0, 0, 0)^T^. Graphical visualization of the typical chaotic ω-limit set associated with dynamical system (5) is given in Figure 13. 

**Figure 14:**
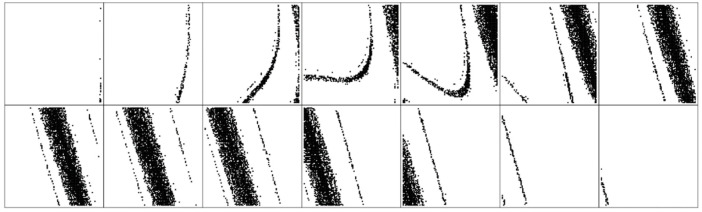
Basin of attraction calculated for dynamical system (4), state space degraded into cubes defined by *x* = 0 and plotted as planes *d*^4^*x*/*dt*^4^ (from left to right), upper row is set {−2, −1, 0, 1, 2, 3, 4}, lower row is continuation {5, 6, 7, 8, 9, 10, 11}.

**Figure 15:**
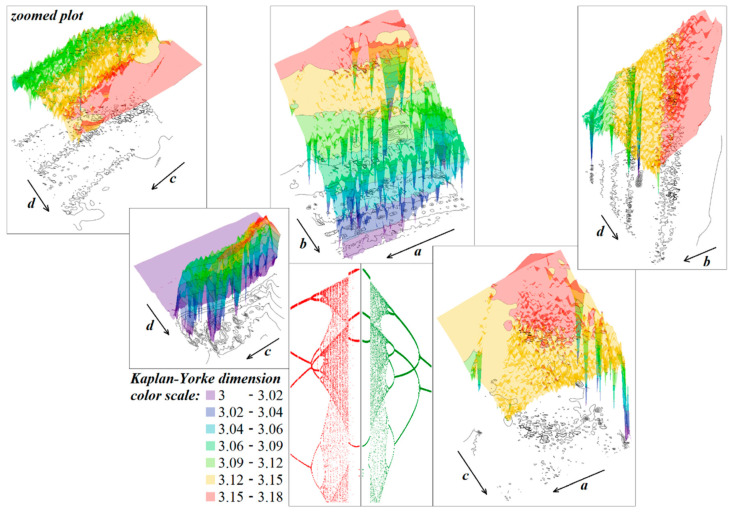
Kaplan–Yorke dimension calculated in close neighborhood of nominal values of internal system parameters, low-resolution one-dimensional bifurcation diagram calculated with respect to the external voltage *V_a_* ∈ (0.4, 0.5)V with a step of 1 mV (red) and *V_b_* ∈ (−0.45, −0.2)V with a step of 1 mV (green).

Figure 14 shows basin of attraction associated with dynamical system (4) calculated for kinetic energy, i.e., if potential energy equals zero. For analyzed fourth-order dynamical system (4), rainbow color-scaled surface contour plots of Kaplan–Yorke metric dimension as two-dimensional function of two internal system parameters are visualized by means of Figure 15. It is shown that the region of strong chaos is surrounded either by the unbounded solution (white) or a weak chaos dynamical motion (green). Note that if the circuit parameters are calculated precisely, the corresponding chaotic oscillators can produce stable chaotic waveforms, both in terms of geometric structure and time. Bifurcation diagrams provided in the same figure demonstrate that a standard period-doubling route-to-chaos scenario can be traced via continuous change of both external voltages. 

### 3.3. Numerical Analysis of Hyperchaotic Dynamics

**Figure 16:**
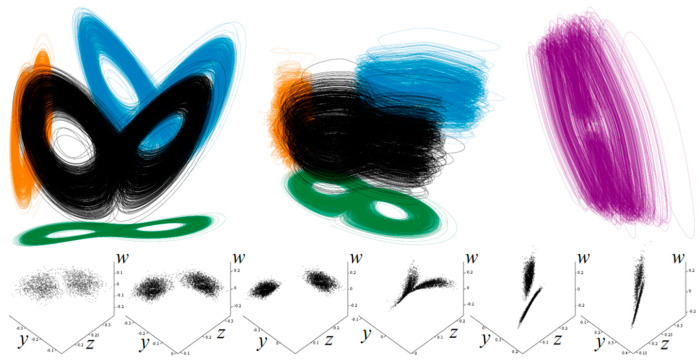
3D perspective plots of typical strange attractor generated by 4D hyperchaotic system (6). Upper left plot: *y* vs. *z* plane (**orange**), *x* vs. *z* plane (**blue**) and *x* vs. *y* plane (**green**). Upper middle plot: *z* vs. *w* plane (**orange**), *y* vs. *w* plane (**blue**) and *y* vs. *z* plane (**green**). Upper right plot: *x* vs. *w* plane (purple). Lower row represents 3D cross sections defined by planes *x* = {−0.25, −0.2, −0.1, 0, 0.1, 0.25}.

**Figure 17:**
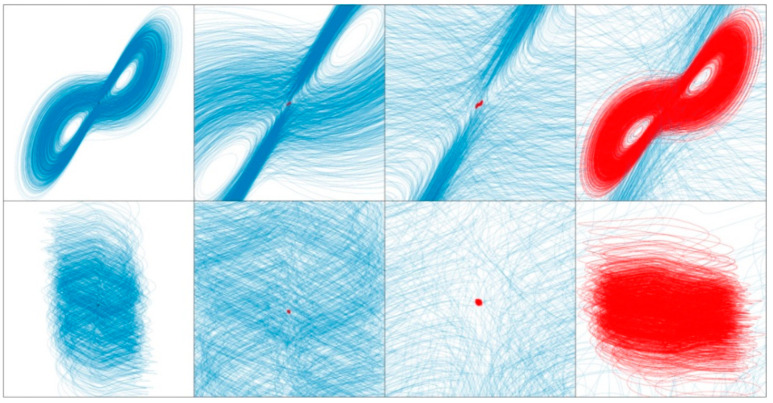
Three-dimensional views of a typical strange attractor associated with 4D hyperchaotic system (6) for increased zoom: original dynamical system (**blue**) and transformed system (**red orbit**). Size of cube side of individual plane projection, from left to right: 50, 10, 4, 0.4.

Hyperchaotic dynamics is specific in the sense that we have two positive Lyapunov exponents. One of the most famous mathematical models capable of producing this kind of dynamical behavior is the so-called fourth-order Lorenz system [59,60]. This system can be described by the following set of first-order ordinary differential equations:

(6) $$\frac{d}{dt}x=\frac{a}{x_{r}}\left(y_{r}\cdot y-x_{r}\cdot x\right)\;\;\;\;\;\;\;\;\;\;\;\;\;\;\;\frac{d}{dt}y=\frac{1}{y_{r}}\left[x\left(b-z_{r}\cdot z\right)x_{r}-y_{r}\cdot y+w_{r}\cdot w\right]\;\frac{d}{dt}z=\frac{1}{z_{r}}\left(x_{r}\cdot y_{r}\cdot x\cdot y-c\cdot z_{r}\cdot z\right)\;\;\;\;\;\;\;\;\;\;\;\;\;\;\;\frac{d}{dt}w=-\frac{d}{w_{r}}x_{r}\cdot x,$$

where **T** = diag(*x_r_*, *y_r_*, *z_r_*, *w_r_*) = (50, 50, 100, 150) represents a fundamental transformation matrix used for strange attractor compression and *a* = 10, *b* = 28, *c* = 2.7, and *d* = 5 are system parameters that lead to hyperchaotic motion. By considering mathematical model (6) and the numerical values mentioned above, we can obtain the strange attractor provided in the different 3D projections by means of Figure 16. During calculations, the final time was set to 1000 s with a fixed time step of 10 ms and initial conditions **x**_0_ = (0, 0, 0, 0.1)^T^. By utilizing the proposed linear change of the state coordinates, the desired state attractor shrinks from unfeasible cube (±20, ±30, +50, ±50) to much smaller cube (±0.4, ±0.5, +0.5, ±0.5). This process is graphically visualized by means of Figure 17. It turns out that even the greater compression of the strange attractor is possible. However, large values of system constants can cause practical problems, both from the viewpoint of trajectory convergence and basin of attraction.

**Figure 18:**
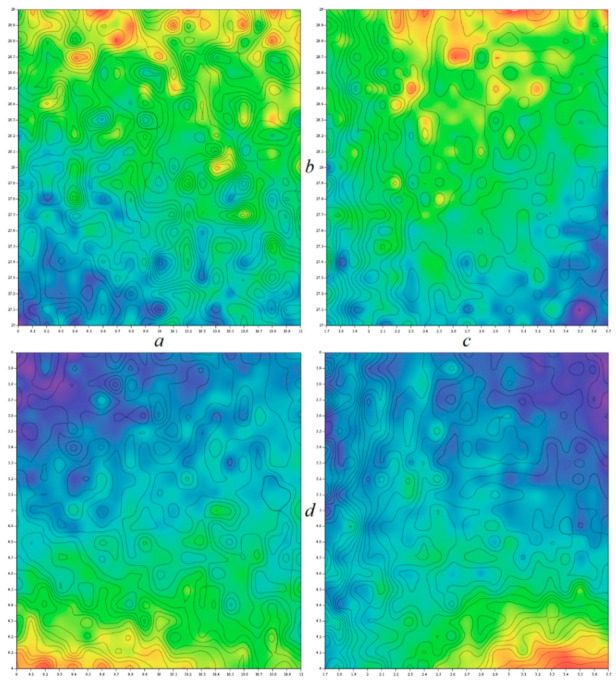
Rainbow color-scaled plots of two LLE as function of internal parameters of hyperchaotic system (6). Colored contours represent maximal LLE while line contours second largest LLE; see text for clarification. Overlapped maxima mark dynamical behavior expanding in two directions.

It is worth nothing that in the case of the dynamical system with four degrees of freedom, just four one-dimensional Lyapunov exponents can be calculated. For chaos, only one LLE is positive and dynamical flow diverges in one state space direction at that moment. However, for hyperchaotic dynamics, two Lyapunov exponents need to be positive. In the hyperspace of internal parameters *a*, *b*, *c*, *d* of system (6), this situation is graphically demonstrated via Figure 18. For this plot, the final time was set to 1000 s, and the initial conditions were randomly selected about the origin. The value of the true LLE is marked by following the color scale: a negative value is blue (fixed point solution), zero is green (limit cycle), while yellow and red are positive LLE (weak and strong chaos). Black contour lines are, in fact, visualization of the second LLE. Thus, where these contour lines show a maximum and this area is red, the inspected system exhibits robust hyperchaotic behavior. Each plot in Figure 18 is calculated as being high-resolution and having 101 × 101 points. Note that if the numerical values provided below Equation (6) are considered, the time domain predictability of the generated signals is not the best and can be further optimized. For basic parameters, the set of Lyapunov exponents is 0.174, 0.132, 0, −15.6, as also indicated in [61]; leading to a Kaplan–Yorke dimension of about 3.02. In this thorough study, a much more detailed linear analysis of the dynamical system (6) is provided.

## 4. New Circuitry Realizations of Chaotic Dynamical Systems Employing DV-TC-M

This section describes the implementation of three different autonomous deterministic dynamical systems as lumped analog circuits. Each case represents a certain type of network synthesis that is straightforward, known, and generally universal but, without the proposed DV-TC-M active device, always requires many active circuit elements. Moreover, common commercially available active devices utilize significantly high supply voltage range, have high power consumption, and exhibit larger parasitic properties. These parasitic features insert error terms into the describing differential equations, change the predefined numerical values of the system parameters, can attenuate important frequency components, can lower complexity of the dynamical solution, and deform or destruct the desired strange attractor. It should be noted that conventional electronic systems such as mixers, harmonic oscillators, modulators, amplifiers, and others can be constructed using single DV-TC-M.

Combination of the differential summing operation with polynomial trans-admittance transfer function allows both building new functional blocks and is interesting from a synthesis perspective (which will be discussed in the upcoming subsection) and optimization of the final oscillator with respect to minimum circuit elements.

### 4.1. Analog Functional Blocks Based on DV-TC-M Active Element

**Figure 19:**
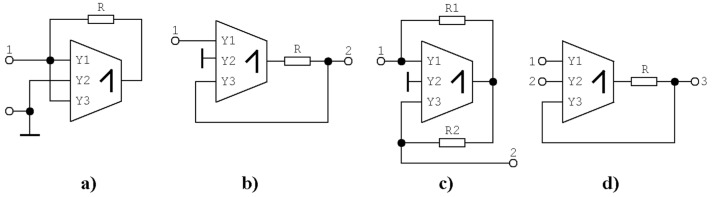
Four examples of differential voltage trans-conductance multiplier (DV-TC-M) subcircuits that are interesting in terms of synthesis of the linear part of the vector field: (**a**) negative grounded resistor, (**b**) unilateral resistor, (**c**) negative trans-admittance two-port, and (**d**) voltage controlled current source.

**Figure 20:**
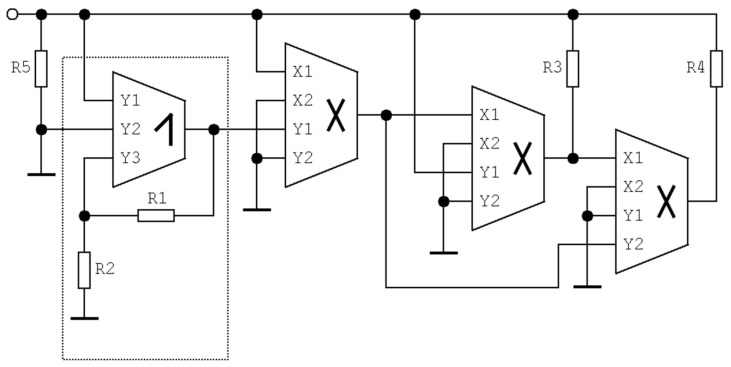
DV-TC-M based sine-type resistor that considers power series approximation with three terms, cosine- type resistor can be implemented by a cascade of three squarers, negative resistor, and constant current source (each can be implemented by single proposed summation/subtraction block).

Thanks to the proposed DV-TC-M active element, we were able to construct several handy linear subcircuits, such as negative grounded resistors (two-port admittance matrix *y*_12_ = *y*_21_ = *y*_22_ = 0, *y*_11_ = −1/*R*), unilateral resistor (with current flowing in single direction, two-port admittance matrix *y*_11_ = *y*_12_ = *y*_22_ = 0, *y*_21_ = 1/*R*), negative bilateral trans-admittance circuit (two-port admittance matrix *y*_11_ = *y*_22_ = 0, *y*_12_ = −1/*R*_1_, *y*_21_ = −1/*R*_2_), differential voltage current source *I_out_* = (*V_Y_*_1_ − *V_Y_*_2_)/*R*, etc. Each mentioned building block is depicted in Figure 19, and can significantly simplify the final network that creates the linear part of the vector field. If speaking about nonlinear elements of chaotic oscillators, the trans-conductance nature of multiplier allows easy implementation of a polynomial resistor by simple connection of input and output terminal. Besides common nonlinear operations such as multiplication, division, squaring, and square rooting (corresponding circuitries can be found in datasheets of almost any commercially available multiplier) developed DV-TC-M allow the user to construct relatively simple resistors with sine-type and cosine-type ampere–voltage characteristics in both signum variants. Change of sign can be realized by reconnection of one input terminal of multiplier; there is no need to change circuit topology. An example of sine-type structure is provided in Figure 20. It is a two-terminal device having input current

(7) $$i=f\left(v\right)=\left(\frac{1}{R_{3}}+\frac{1}{R_{4}}+\frac{1}{R_{5}}\right)v-\frac{k^{2}\alpha }{R_{3}}v^{3}+\frac{k^{3}\alpha^{2}}{R_{4}}v^{5},$$

where the subcircuit in the dotted area is a non-inverting amplifier with gain *α* = 1 + *R*_2_/*R*_1_. Numerical values of all resistors can be calculated by comparing individual terms of (7) with a power series expansion of sine function, that is, sin(*x*) = *x* − *x*^3^/6 + *x*^5^/120. Of course, because this series is finite, approximation is valid only in in the close neighborhood of origin. This is a limitation of the dynamical range.

### 4.2. Chaotic Oscillator with Cyclically Symmetrical Vector Field

**Figure 21:**
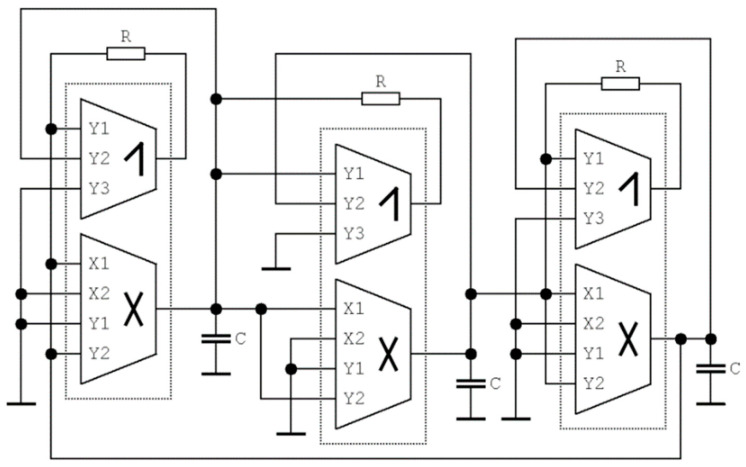
Circuit realization of fully analog chaotic oscillator based on mathematical model having cyclically symmetrical vector field and three pieces of DV-TC-M.

The direct circuitry realization of a dynamical system (3) is provided in Figure 21. Note that only three DV-TC-M active elements are required. Straightforward analysis of this network yields following set of ordinary differential equations

(8) $$C\frac{d}{dt}v_{1}=k_{1}\cdot v_{3}^{2}-\frac{v_{2}}{R}\;\;\;\;\;\;\;\;\;\;C\frac{d}{dt}v_{2}=k_{2}\cdot v_{1}^{2}-\frac{v_{3}}{R}\;\;\;\;\;\;\;\;\;\;C\frac{d}{dt}v_{3}=k_{3}\cdot v_{2}^{2}-\frac{v_{1}}{R},$$

where the state vector **v** = (*v*_1_, *v*_2_, *v*_3_)^T^ is composed of voltages across grounded capacitors taken from left to right, with *k_j_* being the trans-conductance of *j*-th multiplier. The simultaneous change of all resistors (for example, via a tandem potentiometer) varies with system parameter *b* while parameter *a* stays fixed on a constant value given by the impedance norm of this circuit. For experimental verification, capacitors have a uniform value of 100 nF. Experiments show that the time constant of this chaotic oscillator can be lowered; the smallest value of applicable capacitors is 33 pF together with nominal resistance 1 kΩ. The measured power dissipation of this chaotic oscillator lies between 55 and 65 mW; the concrete value depends on the operational regime (chaos vs. limit cycles). 

### 4.3. Circuitry Implementation of Fourth-Order Hyperjerk Function

It is well-known that single third-order differential equations can be easily realized as electronic circuits. The most straightforward approach is a cascade connection of the voltage-mode integrators, two-port linear, and at least one nonlinear feedback [62]. To simplify the final realization, in some cases, the integrator with an active element can be substituted by passive loss realization; i.e., bilinear filter with a single real pole. This kind of realization is not restricted to third-order dynamical systems and can be easily extended to an arbitrary degree of freedom. Circuit realizations of the chaotic systems based on integrator-block schematics can be easily emulated using commercially available field programmable analog array (FPAA) development kits [63,64]. However, remember that the complexity of the implemented chaotic system is limited because the FPAA graphical user interface provides a restricted area dedicated to circuit design. To realize more complex electronic systems, several FPAA kits need to be combined, see [65] for an interesting example where the correct function of the chaotic oscillator is demonstrated including synchronization. 

Unlike other circuitry realization, our solution utilizes only one type of active element, the designed DV-TC-M. Thus, the whole chaotic system except for external working capacitors and resistors can be implemented on chip using the CMOS technology discussed in Section 2 of this paper.

**Figure 22:**
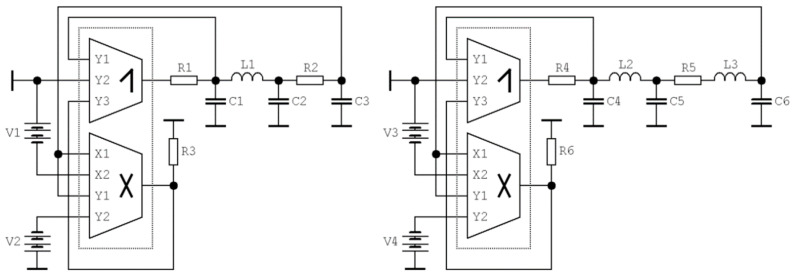
Circuitry implementation of fourth-order (**left schematic**) and fifth-order (**right picture**) jerky dynamical system, both with only a single DV-TC-M active element.

It seems that the simplest practical implementation of mathematical model (2) is based on the looped interconnection of a linear fully passive frequency filter working in the trans-resistance mode (two-port, current input and voltage output) and nonlinear two-port that works in complementary trans-admittance regime. One such example is provided by means of Figure 22; note that the fundamental fourth-order RLC ladder low-pass frequency filter implements the linear part of the vector field. In this case, Equation (2) can be rewritten providing more details as

(9) $$\frac{d^{4}}{dt^{4}}v_{out}+\frac{C_{2}+C_{3}}{C_{2}C_{3}R}\frac{d^{3}}{dt^{3}}v_{out}+\frac{C_{1}+C_{2}}{C_{1}C_{2}L}\frac{d^{2}}{dt^{2}}v_{out}+\frac{C_{1}+C_{2}+C_{3}}{C_{1}C_{2}C_{3}LR}\frac{d}{dt}v_{out}=\frac{1}{C_{1}C_{2}C_{3}LR}f\left(v_{out}\right),$$

where *v_out_* is output voltage of passive filter. Since both the energy source and nonlinearity are created by a single active element (DV-TC-M), the corresponding chaotic oscillator cannot be simplified further. The proposed circuit exhibits a chaotic motion for *C*_1_ = 1 nF, *C*_2_ = 470 pF, *C*_3_ = 820 pF, *L* = 1.8 mH, and *R* = 1.4 kΩ. The state description of this oscillator is

(10) $$C_{1}\frac{d}{dt}v_{1}=k\left(v_{3}-V_{a}\right)\left(v_{3}-V_{b}\right)-i_{L}\;\;\;\;\;\;\;\;\;\;C_{2}\frac{d}{dt}v_{2}=i_{L}-\frac{1}{R}\left(v_{2}-v_{3}\right)\;C_{3}\frac{d}{dt}v_{3}=\frac{1}{R}\left(v_{2}-v_{3}\right)\;\;\;\;\;\;\;\;\;\;L\frac{d}{dt}i_{L}=v_{1}-v_{2},$$

where state vector is **x** = (*v*_1_, *v*_2_, *v*_3_, *i_L_*)^T^ and *k* is internally trimmed constant of DV-TC-M element, namely *k* = 1.3 mA/V^2^. Structurally stable strange attractor can be observed for following list of the circuit parameters: *C*_1_ = 3.9 nF, *C*_2_ = 1.7 nF, *C*_3_ = 4.2 nF, *L*_1_ = 1.8 mH, *R*_1_ = 200 Ω, *R*_2_ = 2500 Ω (both resistances are potentiometers), and *R*_3_ = 750 Ω. External voltages *V_a_* and *V_b_* can be considered as natural bifurcation parameters. Similar concept will be used to design fifth-order chaotic oscillator. In this case, Equation (3) can be rewritten providing more details as

(11) $$\frac{d^{5}}{dt^{5}}v_{out}+\frac{R}{L_{2}}\frac{d^{4}}{dt^{4}}v_{out}+\frac{L_{1}C_{1}\left(C_{1}+C_{3}\right)+L_{2}C_{3}\left(C_{1}+C_{2}\right)}{C_{1}C_{2}C_{3}L_{1}L_{2}}\frac{d^{3}}{dt^{3}}v_{out}+\frac{R_{2}\left(C_{1}+C_{2}\right)}{C_{1}C_{2}L_{1}L_{2}}\frac{d^{2}}{dt^{2}}v_{out}+\;+\frac{C_{1}+C_{2}+C_{3}}{C_{1}C_{2}C_{3}L_{1}L_{2}}\frac{d}{dt}v_{out}=\frac{1}{C_{1}C_{2}C_{3}L_{1}L_{2}}f\left(v_{out}\right),$$

where *v_out_* is output voltage of fifth-order low-pass passive filter. This is the right place to remark that it is possible to substitute trans-impedance RLC passive ladder filter with active low-pass filter with the same, i.e., a trans-impedance type, filter. This holds under specific circumstances, namely in case of the same positions of the transfer function poles in the complex plane. State description of this oscillator can be expressed as

(12) $$C_{1}\frac{d}{dt}v_{1}=k\left(v_{3}-V_{a}\right)\left(v_{3}-V_{b}\right)-i_{L1}\;\;\;\;\;\;\;\;\;\;C_{2}\frac{d}{dt}v_{2}=i_{L1}-\frac{1}{R_{2}}\left(v_{2}-v_{3}\right)\;C_{3}\frac{d}{dt}v_{3}=\frac{1}{R_{2}}\left(v_{2}-v_{3}\right)\;\;\;\;\;\;\;\;\;\;L_{1}\frac{d}{dt}i_{L1}=v_{1}-v_{2}\;\;\;\;\;\;\;\;\;\;L_{2}\frac{d}{dt}i_{L2}=v_{1}-v_{2}-R_{2}i_{L2},$$

where a state vector becomes **x** = (*v*_1_, *v*_2_, *v*_3_, *i_L_*_1_, *i_L_*_2_)^T^ and *k* is a trans-conductance constant of DV-TC-M. In order to generate a structurally stable dense strange attractor, a list of the circuit parameters should be the following: *C*_4_ = 330 pF, *C*_5_ = 390 pF, *C*_6_ = 470 pF, *L*_2_ = *L*_3_ = 1.8 mH, *R*_4_ = 200 Ω, *R*_5_ = 2500 Ω (both resistances are potentiometers). This set is not unique. During experimentation, few other value configurations turn out to be reasonable for the evolution of robust chaos. The time constant of this circuit can be lowered if each capacitor and inductor is divided by the same real number. The analogical change of time constant can be applied in the case of the fourth-order jerky system.

### 4.4. Hyperchaotic System

**Figure 23:**
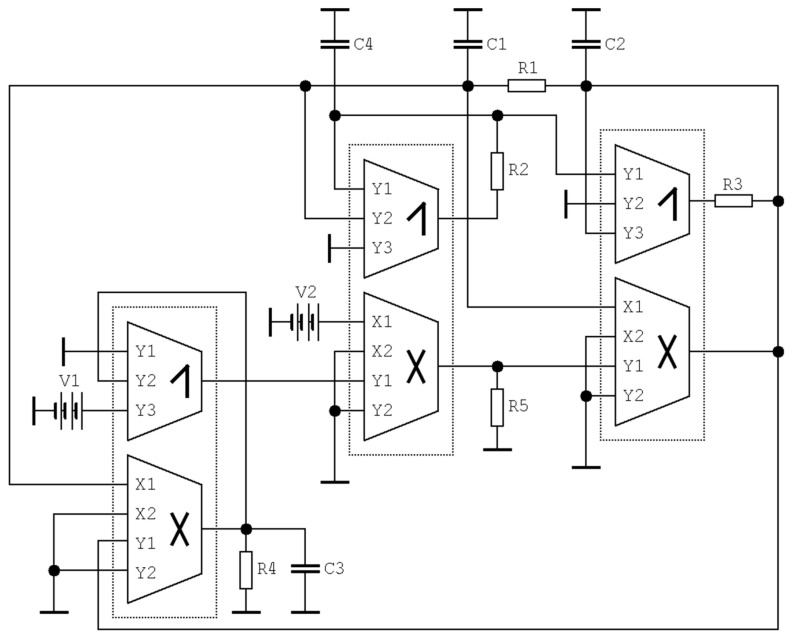
Circuitry realization of a hyperchaotic system with only three DV-TC-M active elements.

Dynamical system (6) represents, from the viewpoint of circuit realization, a complex vector field. Such systems can be implemented by using an analog computer concept [31,35], i.e., a universal method that can be easily applied to almost any mathematical models but employs many active elements. To simplify the final structure, let us assume that individual differential equations describe a sum of currents flowing through a grounded linear capacitor. Considering this, both linear and nonlinear terms of differential equations should work in a trans-admittance regime, i.e., as the current source controlled by input voltage. Thus, the linear part of a vector field can be directly represented by an admittance matrix in terms of the matrix method of the unknown nodal voltages. The design approach based on this synthesis originally dedicated for the circuit analysis can adopt DV-TC-M directly. Assume the following set of ordinary differential equations derived from expression (6)

(13) $$C_{1}\frac{d}{dt}v_{x}=\frac{v_{y}-v_{x}}{R_{1}}\;\;\;\;\;\;\;\;\;\;C_{2}\frac{d}{dt}v_{y}=\frac{v_{x}-v_{y}}{R_{1}}+\frac{v_{w}}{R_{3}}+k_{2}k_{3}R_{5}V_{2}\left(V_{1}-v_{z}\right)v_{x}\;C_{3}\frac{d}{dt}v_{z}=k_{1}v_{x}v_{y}-\frac{v_{z}}{R_{4}}\;\;\;\;\;\;\;\;\;\;C_{4}\frac{d}{dt}v_{w}=-\frac{v_{x}}{R_{2}},$$

where *k_j_* is trans-conductance of *j*-th DV-TC-M multiplier and *V*_1_, *V*_2_ are external DC voltages. This mathematical expression directly corresponds to a circuitry provided in Figure 23. Note that only three DV-TC-M elements are required for design of this chaotic oscillator. By considering unified values of the trans-conductances *k*_1,2,3,4,5_ = 1.3 mA/V^2^, the desired chaotic attractor can be observed if the values of the remaining circuit components are *C*_1_ = 10 nF, *C*_2_ = 5.2 nF, *C*_3_ = *C*_4_ = 100 nF, *R*_1_ = 1 kΩ, *R*_2_ = 600 Ω, *R*_3_ = 333 Ω, *R*_4_ = 4.9 kΩ, *R*_5_ = 50 kΩ, and external voltages *V*_1_ = 290 mV, *V*_2_ = 2 V. The time constant of this circuit is τ = 100 µs, and can be changed by dividing the value of each capacitor by the same number. However, the smallest allowed value of time constant for which the system generated an undistorted shape of a strange attractor is 200 ns. This leads to the set of working capacitors *C*_1_ = 3.2 pF, *C*_2_ = 1.7 pF, *C*_3_ = *C*_4_ = 32 pF, while resistors remain unchanged, and the nominal value equals 1 kΩ.

**Figure 24:**
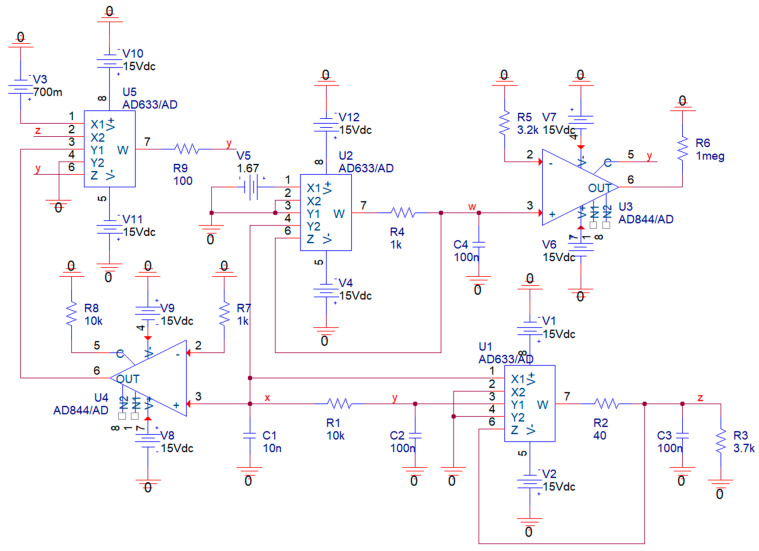
Realization of the hyperchaotic system based on the integrator block schematic ready for OrCAD PSpice circuit simulation, five active devices needed, nodes with the same label are connected, individual capacitors can be pre-charged using pseudo-component IC1 (not mandatory).

**Figure 25:**
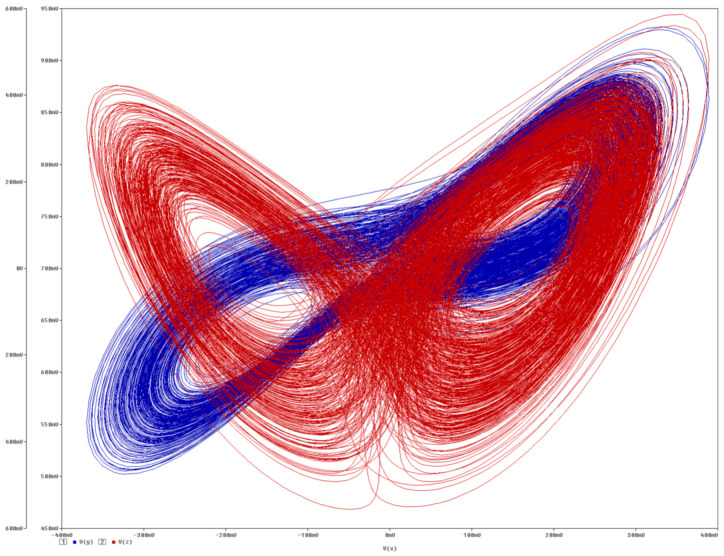

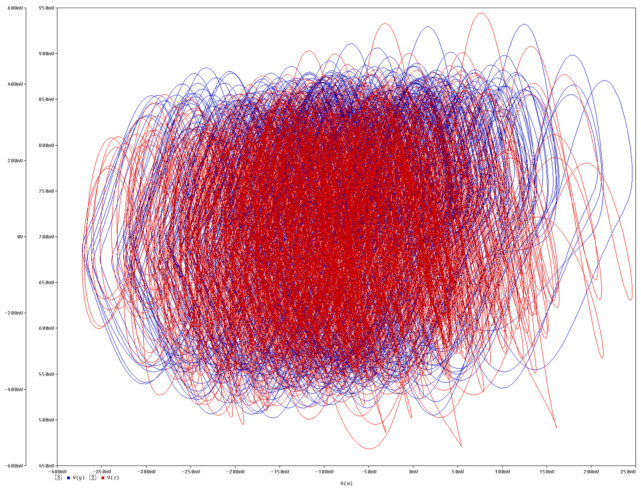
Circuit simulation of hyperchaotic system based on the integrator block schematic. Upper plot: *v_x_* vs. *v_y_* plane (**blue**), *v_x_* vs. *v_z_* plane (**red**). Lower plot: *v_w_* vs. *v_y_* plane (**blue**), *v_w_* vs. *v_z_* plane (**red**).

The dynamical system (6) can be equivalently constructed using the network provided in Figure 24. In this case, describing mathematical model:

(14) $$C_{1}\frac{d}{dt}v_{x}=\frac{v_{y}-v_{x}}{R_{1}}\;\;\;\;\;\;\;\;\;\;C_{2}\frac{d}{dt}v_{y}=\frac{v_{x}-v_{y}}{R_{1}}+\frac{K_{5}}{R_{9}}\left(V_{3}-v_{z}\right)\frac{R_{8}}{R_{7}}v_{x}+\frac{v_{w}}{R_{5}}\;C_{3}\frac{d}{dt}v_{z}=-\frac{v_{z}}{R_{3}}+\frac{K_{1}}{R_{2}}v_{x}v_{y}\;\;\;\;\;\;\;\;\;\;C_{4}\frac{d}{dt}v_{w}=-\frac{K_{2}}{R_{4}}v_{x},$$

where *K_j_* = 0.1 is a non-changeable internal constant of *j*-th fourth-quadrant analog multiplier AD633. Corresponding simulated strange attractor visualized as plane projections are provided by means of Figure 25; numerical values of passive circuit components and the location of symmetrical supply voltage ±15 V are included therein. The time constant of this circuit can be changed by boosting or lowering each capacitor by the same value. However, the smallest value that leads to an undistorted shape of the strange attractor is about 100 pF. 

## 5. Experimental Verification

**Figure 26:**
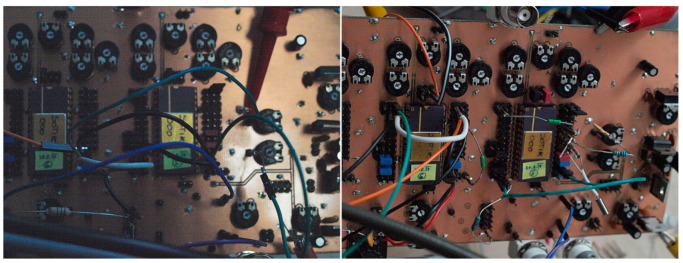
Fabricated printed circuit board (PCB) dedicated to experimental verification of DV-TC-M-based applications: fourth-order jerky function as a robust generator of the chaotic waveforms (**left photo**) and chaotic oscillator with cyclically symmetrical vector field (**right photo**).

**Figure 27:**
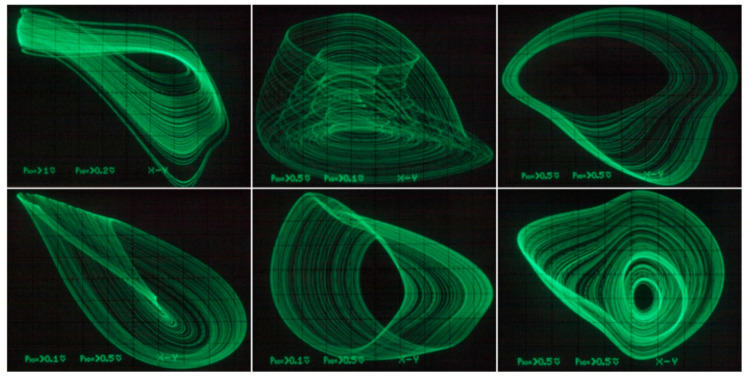
Experimental verification of chaotic circuit given in Figure 21: selected plane projections.

**Figure 28:**
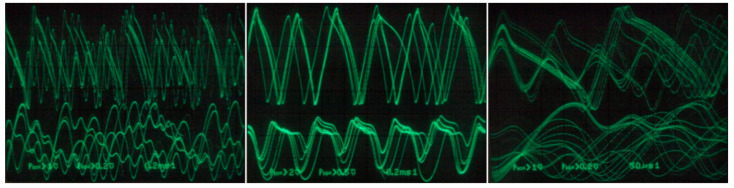
Experimental verification of chaotic circuit given in Figure 21: generated chaotic signals.

Individual chaotic oscillators based on DV-TC-M active element have been implemented using the universal breadboard visualized in Figure 26. True experimental results associated with a dynamical system (7) are provided by means of Figure 27 (different Monge projections of a chaotic state orbit) and Figure 28 (chaotic waveforms in different time scales). 

**Figure 29:**
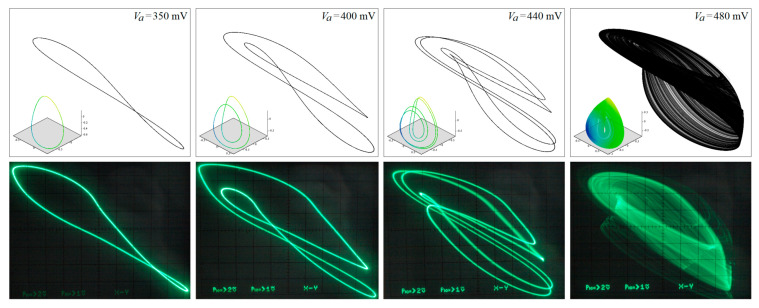
Outputs coming from the numerical integration process (**upper row of plots**) compared to true experimental results captured as the oscilloscope screenshots: *v*_1_ vs. *v*_2_ plane projections of the generated state attractors (including chaotic orbits), different values of the external voltage *V_a_* and fixed voltage *V_b_* = 350 mV.

**Figure 30:**
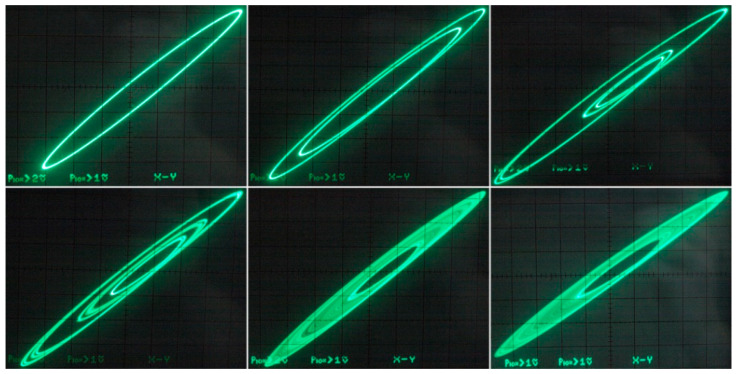
Experimental results captured as the oscilloscope screenshots: *v*_1_ vs. *v*_3_ plane projections of generated state attractors (including chaotic orbits), different values of external voltage *V_a_* and fixed voltage *V_b_* = 350 mV.

**Figure 31:**
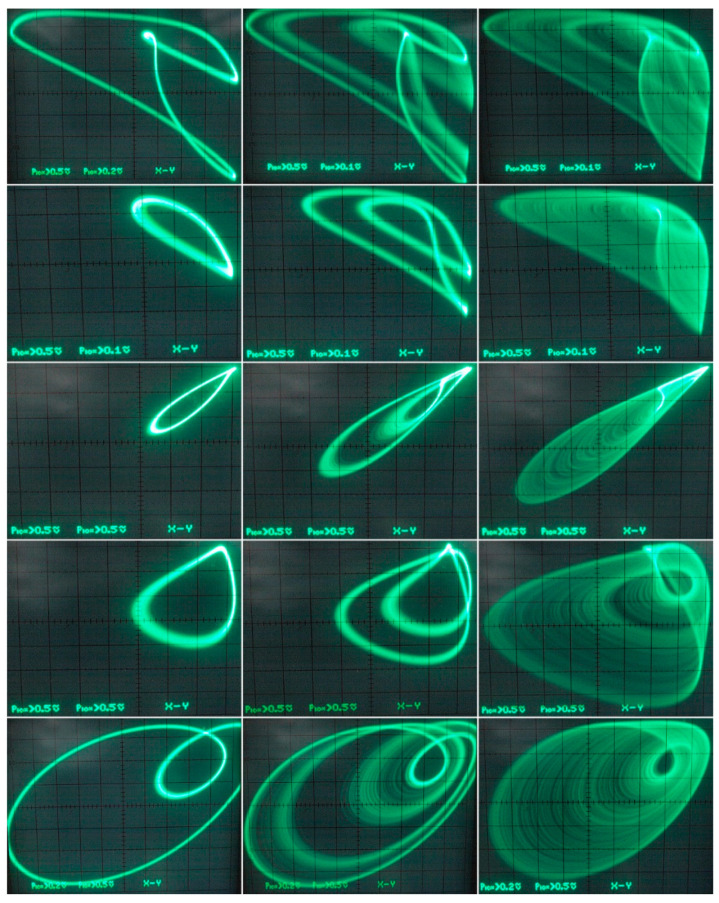
Oscilloscope screenshots captured during measurement of fourth-order jerky dynamics, individual rows represent different plane projection of the limit cycles and typical chaotic attractors.

**Figure 32:**
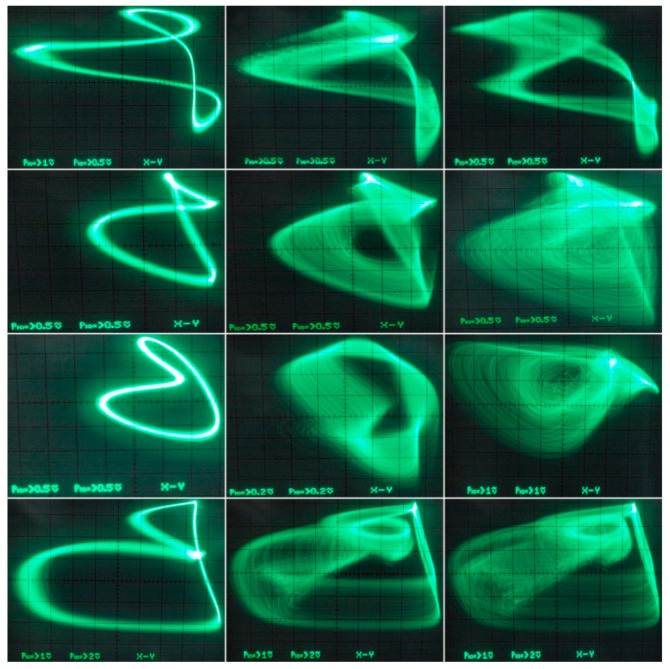
Oscilloscope screenshots captured during verification of a fifth-order jerky chaotic circuit, plane projections of different limit cycles (left column) and chaotic motion (the rest of photos).

**Figure 33:**
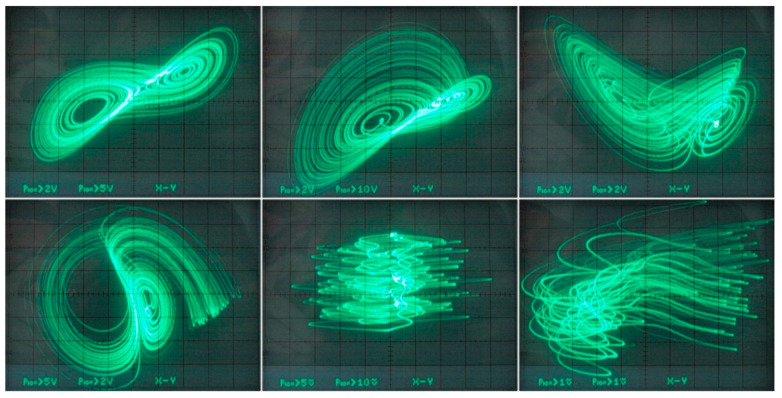
Oscilloscope screenshots associated with measurement of a hyperchaotic circuit, different plane projections of the compressed strange attractor.

Figure 29 demonstrates a very good agreement between theoretical expectations and practical measurements. Figure 30 provides the observed route to chaos scenario via period doubling bifurcation scenario for fourth-order jerky dynamics. Figure 31 shows the family of limit cycles and the consequent strange attractors associated with the fourth-order jerky system given in Figure 22 on the left schematic. Figure 32 provides analogical results for a fifth-order jerky oscillator provided in Figure 22 on the right schematic. Finally, the experimental confirmation of hyperchaos that evolves in the designed fully analog oscillator is provided in Figure 33. Again, the shape of the measured strange attractor is closely related to that numerically integrated; see Figure 16.

## 6. Discussion

This paper brings detailed description of the integrated bipolar/CMOS design of a new active element denoted-by-authors as DV-TC-M that is optimized from the viewpoint of circuit modeling of the smooth vector fields. The major advantages can be summarized as follows: it comprises cumulated mathematical operations, accurate polynomial transfer function, satisfactory dynamical range, low long-term vulnerability of intrinsic parameters, high roll-off frequencies associated with important transfer functions, and voltage-input and current-output (for both multipliers). The feasibility of the proposed CMOS structure is demonstrated in several different design scenarios of a fully analog chaotic oscillator. We can conclude perfect one-to-one correspondence between theoretical expectations (i.e., analysis of mathematical model) and practical experiments (captured oscilloscope screenshots). All circuitry realizations utilize the minimal number of passive elements and simultaneously save several active devices if the final circuit is compared to a conventional synthesis method, such as analog computing [66]. Since the design of lumped chaotic oscillators still represents an up-to-date topic, this paper leaves place for future research.

Recently, the attention of mathematicians, physicians, and analog design engineers has been attracted to so-called fractional-order (FO) dynamical system modeling. FO differential equations are used to reach better accuracy in the description of some specific phenomena, and can be used in the mathematical model of chaotic dynamics [67,68,69,70]. Since non-integer order derivation is bounded to the accumulation element, DV-TC-M active elements can also be utilized to simplify construction of the FO chaotic oscillators. Remember that FO two-terminal devices need to be approximated in a wide frequency range, simply because the spectrum of chaotic signals is continuous and wideband. For possible passive ladder structures and detailed design method, consult [71]. The contribution of developed DV-TC-M to the synthesis of the FO circuit element itself (new structures, advantages, parameters) is still uncovered.

In the end, a brief comparison between the proposed network solutions with chaotic oscillators fully integrated on chip needs to be mentioned. Firstly, DV-TC-M is a real active element that can be used in any concept of lumped chaotic oscillator or circuit model of any dynamical system. On the other hand, the existence of external elements does not contribute positively to the accuracy of modeling. Of course, chaotic circuits having only DV-TC-M devices composed by CMOS multiplier and summation/subtraction unit can be considered fully integrated. Such configurations can be, at least in some of its typical parameters, compared with other fully integrated chaotic systems. For example, [72] presents an integrated generator of multidirection spiral attractors. The complexity of this circuit is comparable with the hyperchaotic circuit presented in Figure 23. As expected, a fully integrated oscillator occupies less chip area; only 0.177 mm^2^ is reported while three DV-TC-M devices alone will occupy an area of 0.242 mm^2^. The authors of [72] announced an operational power consumption of 99.5 mW (simulation), and this is value is close to the measured power dissipation for the proposed hyperchaotic circuit. The hyperchaotic oscillator implemented on a chip is the subject of [73]. The total chip area occupied by this circuit is 0.69 mm × 0.84 mm, and this value is still less than the area potentially required by our network topology. However, power consumption is significantly high; up to 475 mW was reported. Interestingly, on-chip realization of a multigrid spiral attractor generator is presented in [74]. The design process is nicely described though the oscillators are based on a conventional electronically programmable second-generation current conveyors, and the vector field is piecewise linear. The authors report excellent frequency responses, while the chip area needed is a little bit larger, and static power consumption (for the simplest configuration of spirals) is about 3.7 mW. This value can be compared to the power dissipation of our generator of a single-scroll attractor, that is, fourth-order jerky dynamics provided in Figure 22. Here, the measured value is approximately four times greater. Another successful realization of multiscroll attractor generator can be found in paper [75]. Of course, a list of the available publications dealing with integrated versions of chaotic systems (the most often fabricated using CMOS technology) is by no means complete. A few others can be found in [76,77], where complete simulations can be found, including a post-layout and process–voltage–temperature variations. However, these results cannot be directly compared and discussed in the context of individual circuitry realizations presented in this paper. Roughly speaking, a fully integrated fashion of implementation of simple as well as complex chaotic oscillators is preferred if portable systems are required for applications.

## 7. Conclusions

This paper introduces a novel active device dedicated not only for the realization of chaotic electronic systems but for continuous time signal processing in general. The credibility of DV-TC-M is demonstrated by experimental verification of three deterministic dynamical systems with complex polynomial vector fields implemented transparently as the simple (much simpler than equivalent structures composed by the conventional active elements) lumped chaotic oscillators. The captured oscilloscope screenshots demonstrate excellent agreement with numerical results. The calculated values of LLE imply potential applications for the designed chaotic circuits in radio frequency signal processing functional blocks, such as masking, modulation, and cryptography.

## Figures and Tables

**Figure A1:**
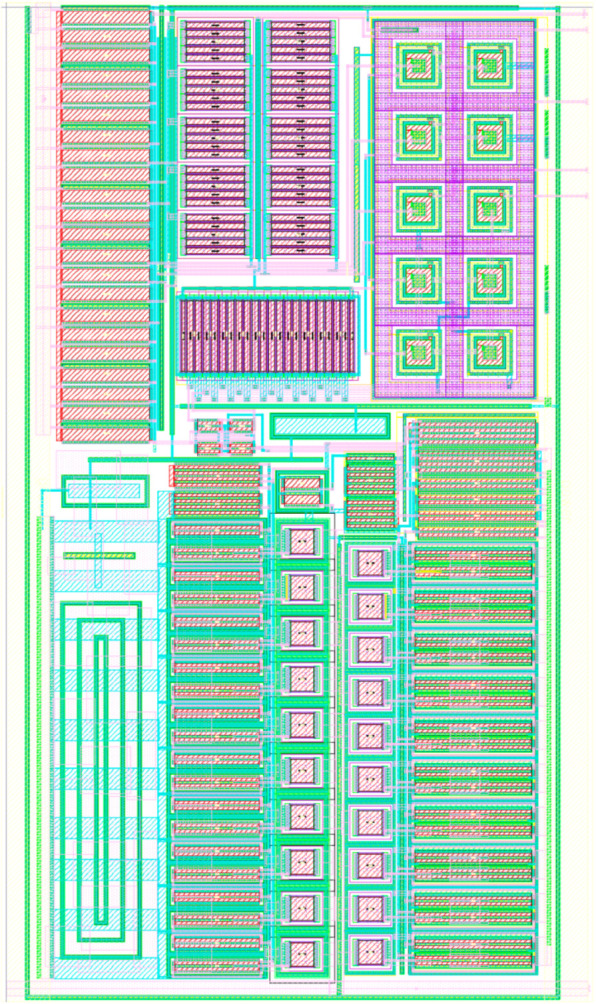
Layout design of multiplier (Cadence Virtuoso IC6) in bipolar technology.

**Figure A2:**
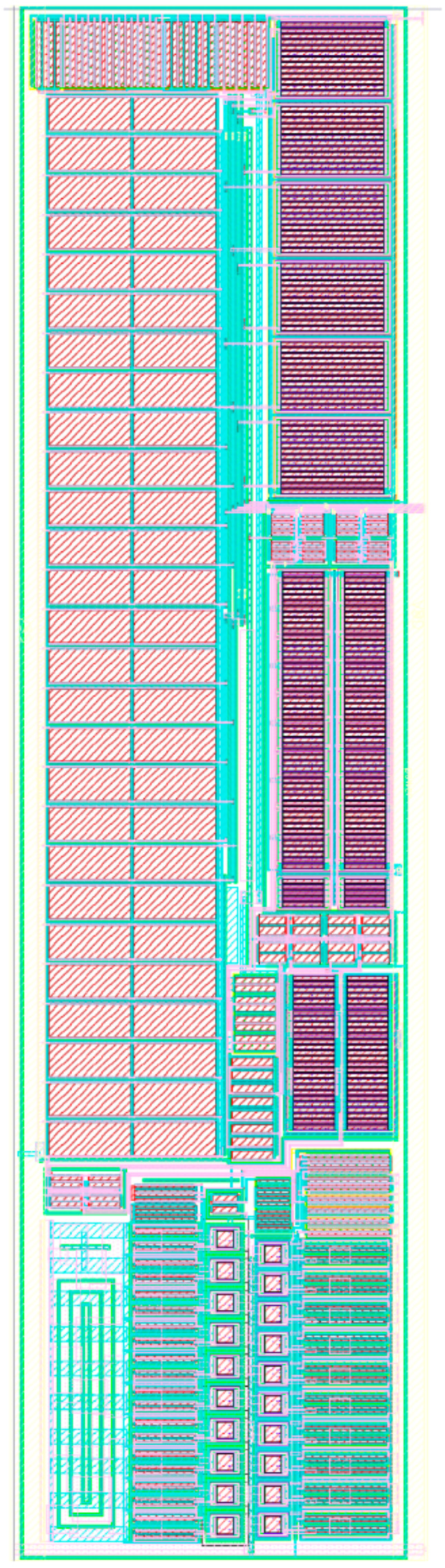
Layout design of multiplier (Cadence Virtuoso IC6) in CMOS technology.

**Figure A3:**
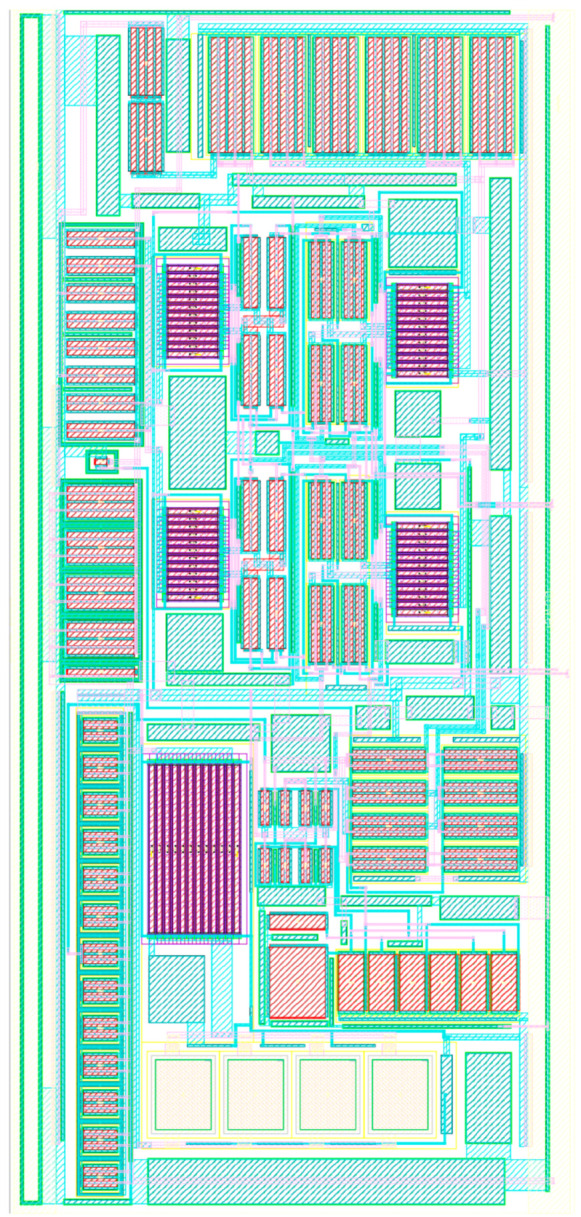
Layout of summation/subtraction cell (Cadence Virtuoso IC6) in CMOS technology.

## Appendix A

This appendix contains images showing the layouts of individual designed integrated functional blocks: bipolar trans-conductance mode multiplier is in Figure A1, CMOS trans-conductance mode multiplier is provided in Figure A2, and voltage mode summation/subtraction cell is given in Figure A3. Mentioned analog building blocks occupy the following chip areas: 340 µm × 203 µm = 0.07 mm^2^ (bipolar multiplier), 761 µm × 203 µm = 0.155 mm^2^ (CMOS multiplier) and 430 µm × 203 µm = 0.087 mm^2^ for voltage summation/subtraction cell. Bias power consumption is: 10 mW (bipolar multiplier), 8 mW (CMOS multiplier), and 9 mW (voltage summation/subtraction cell).
